# A Missense Mutation in CRYBB2 Leads to Progressive Congenital Membranous Cataract by Impacting the Solubility and Function of βB2-Crystallin

**DOI:** 10.1371/journal.pone.0081290

**Published:** 2013-11-28

**Authors:** Weirong Chen, Xiaoyun Chen, Zhengmao Hu, Haotian Lin, Fengqi Zhou, Lixia Luo, Xinyu Zhang, Xiaojian Zhong, Ye Yang, Changrui Wu, Zhuoling Lin, Shaobi Ye, Yizhi Liu, for the study group of CCPMOH

**Affiliations:** 1 State Key Laboratory of Ophthalmology, Zhongshan Ophthalmic Center, Sun Yat-sen University, Guangzhou, People's Republic of China; 2 State Key Laboratory of Medical Genetics, Xiangya Medical College, Central South University, Changsha, People's Republic of China; 3 Zhongshan School of Medicine, Sun Yat-sen University, Guangzhou, People's Republic of China; Case Western Reserve University, United States of America

## Abstract

Congenital cataract is a major cause of visual impairment and childhood blindness. The solubility and stability of crystallin proteins play critical roles in maintaining the optical transparency of the lens during the life span. Previous studies have shown that approximately 8.3%∼25% of congenital cataracts are inherited, and mutations in crystallins are the most common. In this study, we attempted to identify the genetic defect in a four-generation family affected with congenital cataracts. The congenital cataract phenotype of this four-generation family was identified as membranous cataract by slit-lamp photography. Mutation screening of the candidate genes detected a heterozygous c.465G→C change in the exon6 of the βB2-crystallin gene (CRYBB2) in all family members affected with cataracts, resulting in the substitution of a highly conserved Tryptophan to Cystine (p.W151C). The mutation was confirmed by restriction fragment length polymorphism (RFLP) analysis and found that the transition resulted in the absence of a BslI restriction site in the affected members of the pedigree. The outcome of PolyPhen-2 and SIFT analysis predicted that this W151C mutation would probably damage to the structure and function of βB2-crystallin. Wild type (wt) and W151C mutant βB2-crystallin were expressed in human lens epithelial cells (HLECs), and the fluorescence results showed that Wt-βB2-crystallin was evenly distributed throughout the cells, whereas approximately 34.7% of cells transfected with the W151C mutant βB2-crystallin formed intracellular aggregates. Taken together, these data suggest that the missense mutation in CRYBB2 gene leads to progressive congenital membranous cataract by impacting the solubility and function of βB2-crystallin.

## Introduction

Congenital cataract, the loss of eye lens transparency, is one of the common causes of visual impairment and childhood blindness. It has an estimated incidence of 1-6 per 10,000 live births [1]. Congenital cataract is particularly serious because it has the potential for inhibiting visual development, resulting in permanent blindness. Approximately 8.3%∼25% of congenital cataracts are inherited, of which autosomal dominant inheritance is the most common. To date, over 20 genes have been identified responsible for isolated autosomal dominant congenital cataract [2]. It has been reported that about half of mutations in crystallins and a quarter in connexins (gap junction proteins), with the remainder divided among the genes for heat shock transcription factor-4 (HSF4), aquaporin-0 (AQP0, MIP), and beaded filament structural protein-2 (BFSP2) [2], [3].

Crystallin proteins, including α-, β- and γ-crystallins, represent about 90% of lens soluble proteins in human. The solubility and stability of these proteins play critical roles in maintaining the optical transparency and high refractive index of the lens during the life span. The α-crystallins are large protein complexes in the lens composed of αA- and αB-crystallins. In addition to their structural roles, α-crystallins are members of the small heat shock protein family and exhibit important molecular chaperone activity within the lens[4]. The β- and γ-crystallins are recognized as members of a related β/γ-crystallin superfamily. They are the predominant structural proteins and play key roles in the development of the lens[5]. β-crystallins are the most abundant water-soluble proteins in the lens and most expressed in lens cortical fiber cells. βB2 is the major β-crystallin in the lens and the least modified during aging [6]. It is also the most thermally stable and soluble of all the β-crystallins, remaining soluble during aging and is needed to maintain the solubility of hetero-oligomers during isolation [7]. There is a tendency for other β-crystallins to precipitate when separated from βB2. This has led to the proposal of a role for βB2 in maintaining the solubility of other crystallins that are heavily modified during aging [8].

About half of the mutations which have been identified responsible for autosomal dominant congenital cataract are in the crystallins genes [2]. So far, fourteen mutations have been reported in CRYBB2, all of them in families with autosomal dominant cataract formation [2], [9]-[15]. The cataract phenotypes reported with mutations in the βB2-crystallins in each family is very different despite the identical mutation, indicating that other modifier genes are likely to influence the cataract phenotype [16]. Previous study has demonstrated that most mutations in the β-crystallins would cause protein structure abnormality, resulting in an unstable protein that precipitates from solution and serves as a nidus for additional protein denaturation and precipitation, eventually resulting in cataract formation [17].

In this study, we attempted to identify the genetic defect in a four-generation family affected with congenital membranous cataracts. We reported the identification of a missense mutation in exon 6 of CRYBB2 that led to an exchange of Trp for Cys (W151C) as the probable cause of the disease in this family. The mutant, W151C of βB2-crystallin would damage to the solubility of βB2-crystallin and result in the formation of aggregates in human lens epithelial cells (HLECs). Although the identical mutation was reported in an Indian family with congenital central nuclear cataract, the phenotype is very different.

## Materials and Methods

### Clinical evaluation and DNA specimens

A 4-generation Chinese family with autosomal dominant congenital cataract was enrolled from the Childhood Cataract Program of the Chinese Ministry of Health (CCPMOH), which includes a series of ongoing studies on the influence of early interventions on long-term outcomes of pediatric cataract treatment [18]. The study was approved by the ethics committee of Zhongshan Ophthalmic Center, Guangzhou, China. Written informed consent was obtained from all participants or their guardians and the study protocol adhere to the tenets of the Declaration of Helsinki. In total, 59 individuals participated in the study, 20 affected and 39 unaffected. Cataracts in affected individuals were either present at birth or developed during childhood. All participants underwent ophthalmologic examination, including visual acuity, intraocular pressure, slit-lamp examination and fundus examination with the dilated pupils. Phenotypes were documented by slit lamp photography. Clinical data on the affected members were obtained from surgical case history records. Blood samples were collected and leukocyte genomic DNA was extracted using QIA amp DNA kit (Qiagen, Valencia, CA, USA).

### Mutation screening

Genomic DNA samples from affected and unaffected members of the family were screened for mutations in CRYAA, CRYAB, CRYBB1, CRYBB2, CRYBB3, CRYGC, CRYGD, CRYGS, GJA3 and GJA8 by directly sequencing. The isolated DNA was amplified by polymerase chain reaction (PCR) for the exons and their flanking regions using previously published primers sequences with modification for exon 6 of the CRYBB2 [10], [12], [19]. Primers for exon 6 of the CRYBB2 were as follows: forward; 5′-AGAAAGCAGAGGCTCAGTGC -3′ and reverse; 5′-GGAGATCAAAGACCCACAGC-3′. The PCR products were sequenced from both directions with the ABI BigDye Terminator cycle sequencing kit v3.1 on a genetic analyzer (ABI Applied Biosystems, Foster City, CA) on an ABI PRISM 3730 Sequence Analyzer (ABI). Variations were identified by importing the sequencing results from patients and consensus sequences from the NCBI human genome database into the SeqManII program of the Lasergene package (DNAStar Inc.,Madison, WI).

### Restriction fragment length polymorphism analysis

After identifying a mutation in exon 6 of the CRYBB2 gene, all family members were confirmed by restriction fragment length polymorphism (RFLP) analysis. The mutation resulted in the absence of cleavage sites for the restriction enzyme BslI in affected family members. PCR products of exon 6 of the CRYBB2 gene were digested for 3 h at 55°C with Bsl I (TAKARA, Dalian, China), and then electrophoresized in 2% agarose gels and analyzed under ultraviolet light.

### Bioinformatics analysis

To determine whether the specific amino acid substitution in the protein sequence might lead to altered the protein function and possibly contribute to the disease, the PolyPhen-2 (Polymorphism Phenotyping) program (http://genetics.bwh.harvard.edu/ggi/pph2/, provided in the public domain by the Harvard Medical School, Boston, MA) and Sorting Intolerant Form Tolerant (SIFT, http://sift.jcvi.org/, provided in the public domain by the J. Craig Venter Institute, CA) were used to predict the possible impact of the amino acid substitution on the structure and function of βB2-crystallin. The prediction is based on the position-specific independent counts score derived from multiple sequence alignments of observations. The prediction outcome of PolyPhen-2 includes benign, possibly damaging and probably damaging to protein function, while the outcome of SIFT score ranges from 0 to 1. The amino acid substitution is predicted damaging if the score is < =  0.05, and tolerated if the score is>0.05.

### Cell culture and transfection

The human lens epithelial cell (HLECs) line HLEB3 was cultured at 37°C in Dulbecco's modified Eagle's medium (DMEM) supplemented with 10% fetal bovine serum (FBS) in a humidified atmosphere containing 5% CO_2_. For fluorescence microscopic imaging, cells were seeded on cover slips in 6-well plates one day before transfection. HLECs were transfected with 2 µg of plasmids that express wt-βB2-crystallin-GFP or the mutant. Transfections were carried out using complete growth medium and TurboFectin 8.0 transfection reagent (OriGene, Rockville, MD) at a 3∶1 TurboFectin 8.0 to DNA ratio for 48 h.

### Immunofluorescence microscopy for aggregates detection

The transfected cells were washed with PBS and fixed with acetone for 10 min at room temperature. The cells were washed with PBS three times and stained with DAPI for 5 min to stain nuclei. The slides were mounted with anti-fade mounting medium and observed under a fluorescence confocal microscope (LSM510; Carl Zeiss, Overkochen, Germany). To quantify the number of transfected cells with aggregates, transfected cells showing aggregates were counted in five fields at 40×magnification. Fields were randomly chosen and contained about 30 cells per field. Experiments were repeated three times and counts were blindly performed.

## Results

### Clinical data

**Figure 1:**
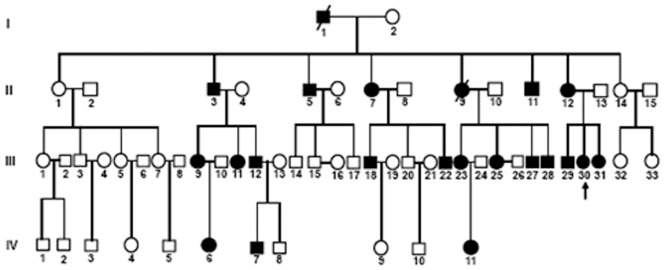
Family pedigree. The family history reveals 22 affected members in 4 generations. The dark symbols represent the affected members of the family, while the clear symbols indicate the healthy ones. Squares and circles indicate males and females respectively. The proband is marked with an arrow. The pedigree of the family suggests an autosomal dominant mode of inheritance.

**Figure 2:**
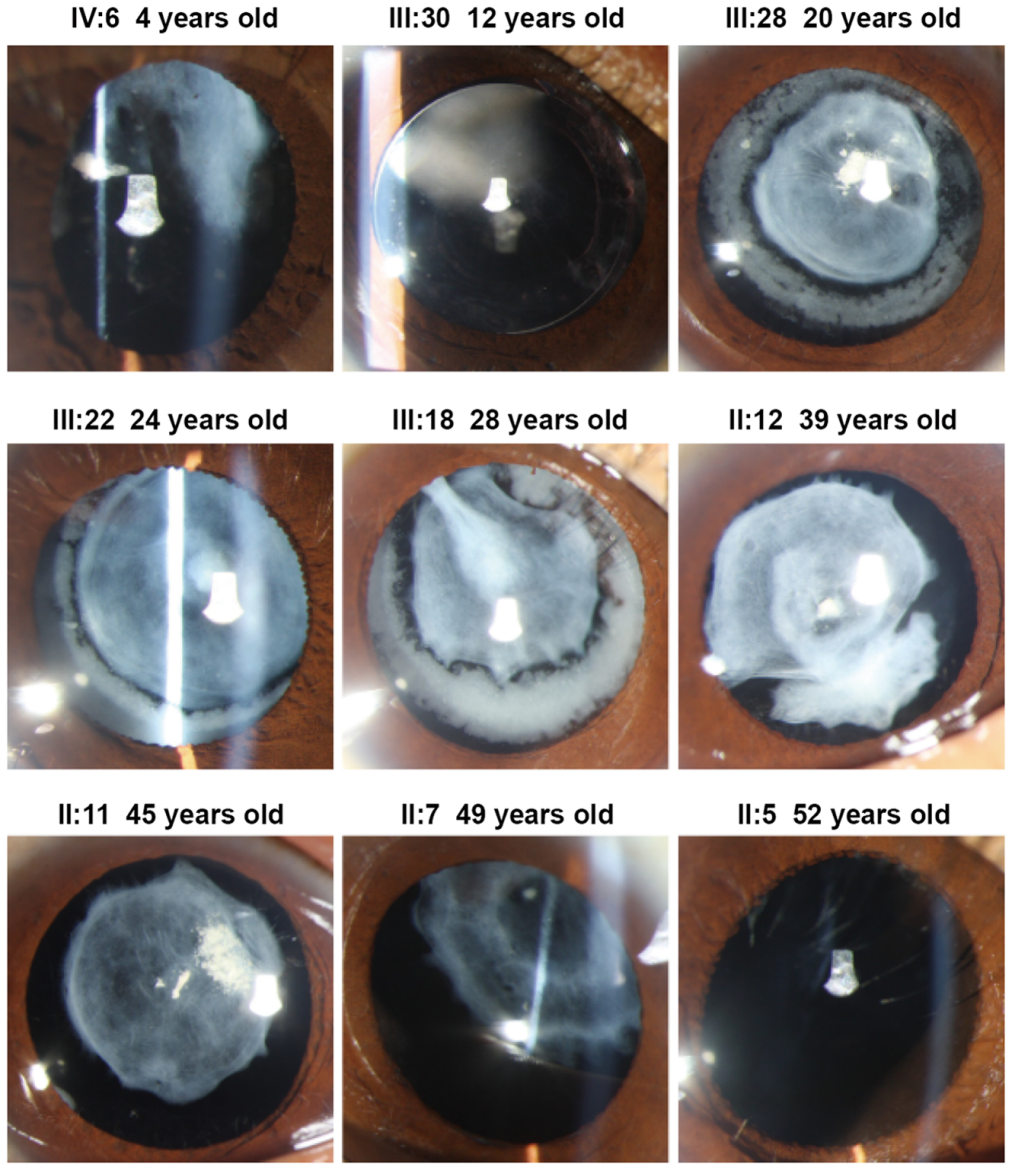
Clinical features of the family. Slit-lamp photographs of affected individuals show the phenotype of congenital cataract is membranous cataract. The lens opacities became denser and upward dislocation gradually with increasing age. In addition, the lens cortex was dissolved gradually as age increased. Over 50 years old, the lens cortex could be dissolved completely.

This 4 generation family included 22 affected individuals with congenital membranous cataract and 39 unaffected individuals. The cataract exhibited an autosomal dominant inheritance pattern in the family. A pedigree of the family is given in Figure 1. The proband (III: 30), a 12-year-old girl, whose mother, sister and brother also suffered from congenital cataract from birth. Twenty affected individuals were examined and available for this study. Opacification of the lens were bilateral in all affected individuals. Visual acuity in the affected individuals before surgery ranged from 0.01 to 0.8. Most affected individuals noticed their visual impairments before the age of ten, and their visual acuity decreased gradually until surgery. As shown in the Figure 2, the phenotype of congenital cataract was membranous cataract. We found that the lens opacities became denser and upward dislocation gradually with increasing age. In addition, the lens cortex was dissolved gradually as age increased because of the rupture of capsules. Over 50 years old, cortex of the lens could be dissolved completely. Apart from congenital cataract, the proband, one female and two girls (II: 7, IV: 6 & IV: 11) also presented with strabismus. No other ocular or systemic abnormalities were found upon physical examination in the affected individuals.

### Mutation screening

**Figure 3:**
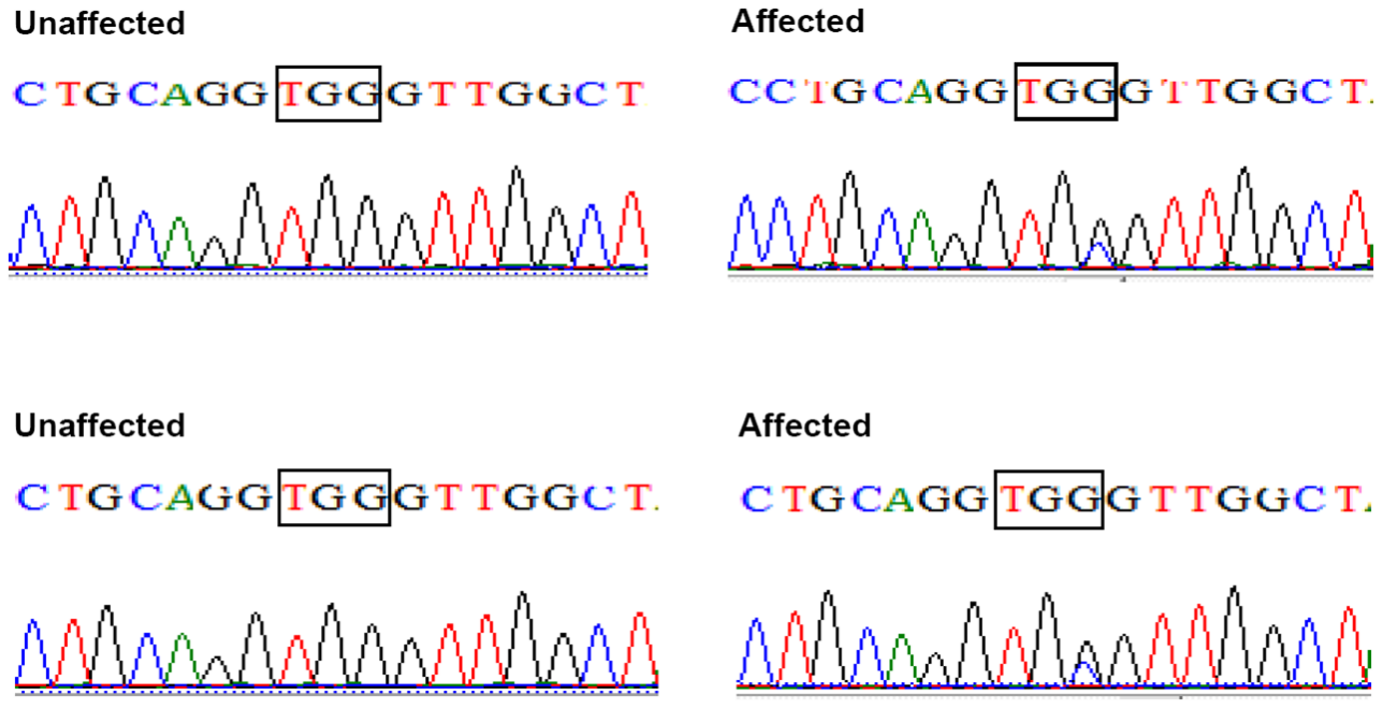
Mutation screening. Forward sequence analysis of the normal and affected sequence of exon 6 of the CRYBB2 gene in this Chinese family. The sequence chromatogram indicates that there is a heterozygous mutation (c.465G>C) in exon 6 of CRYBB2 (black triangles), which leads to the replacement of Tryptophan with Cystine at the 151 amino acid position (p. Trp151Cys).

Bidirectional sequencing of the coding regions of the candidate genes showed only one heterozygous change in exon 6 (G>C) of CRYBB2, at position c.465 of the CRYBB2 gene in all twenty affected family members (Figure 3), leading to the replacement of a highly conserved Tryptophan with Cystine at the 151 amino acid position (p. Trp151Cys). This substitution was not seen in the unaffected individuals of the family or in the 100 unrelated control subjects from the same Chinese population (data not shown). No sequence variation was identified in the coding regions of the CRYAA, CRYAB, CRYBB1, CRYBB3, CRYGC, CRYGD, CRYGS, GJA3 and GJA8 genes.

### Restriction fragment length polymorphism analysis

**Figure 4:**
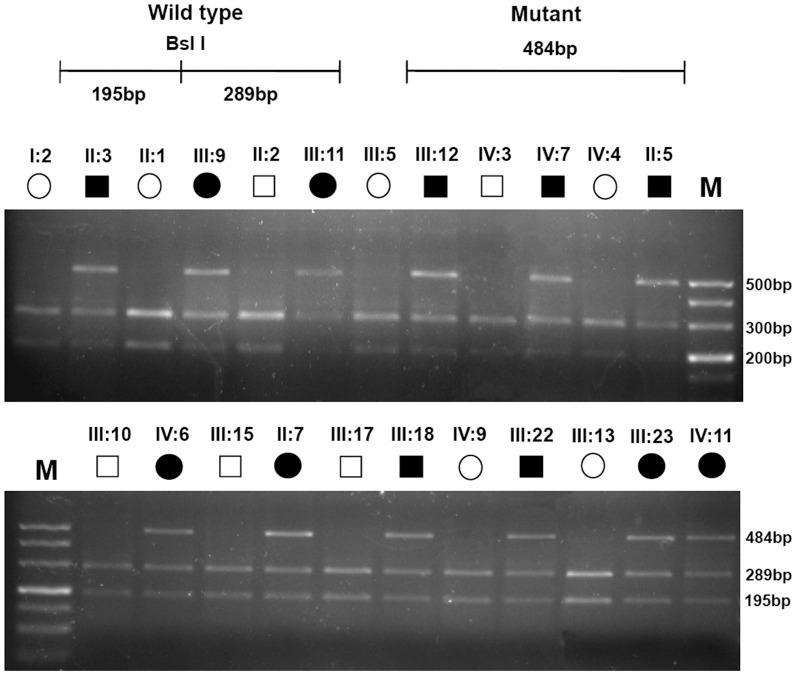
Restriction fragment length polymorphism analysis. RFLP analysis shows that a loss of the BslI restriction site in all the affected individuals heterozygous with the W151C mutation (195, 289 and 484 bp), but was not detected in the unaffected individuals (195 and 289 bp). 500 bp DNA ladder was used as size standard.

The mutation was confirmed by a BslI digest of the PCR amplified exon 6 of CRYBB2 gene. This mutation resulted in the absence of a BslI restriction site in all the affected members of the family, but was not detected in the unaffected pedigree members (Figure 4).

### Bioinformatic evaluation of the impact of W151C mutation on the structure and function of βB2-crystallin

**Figure 5:**
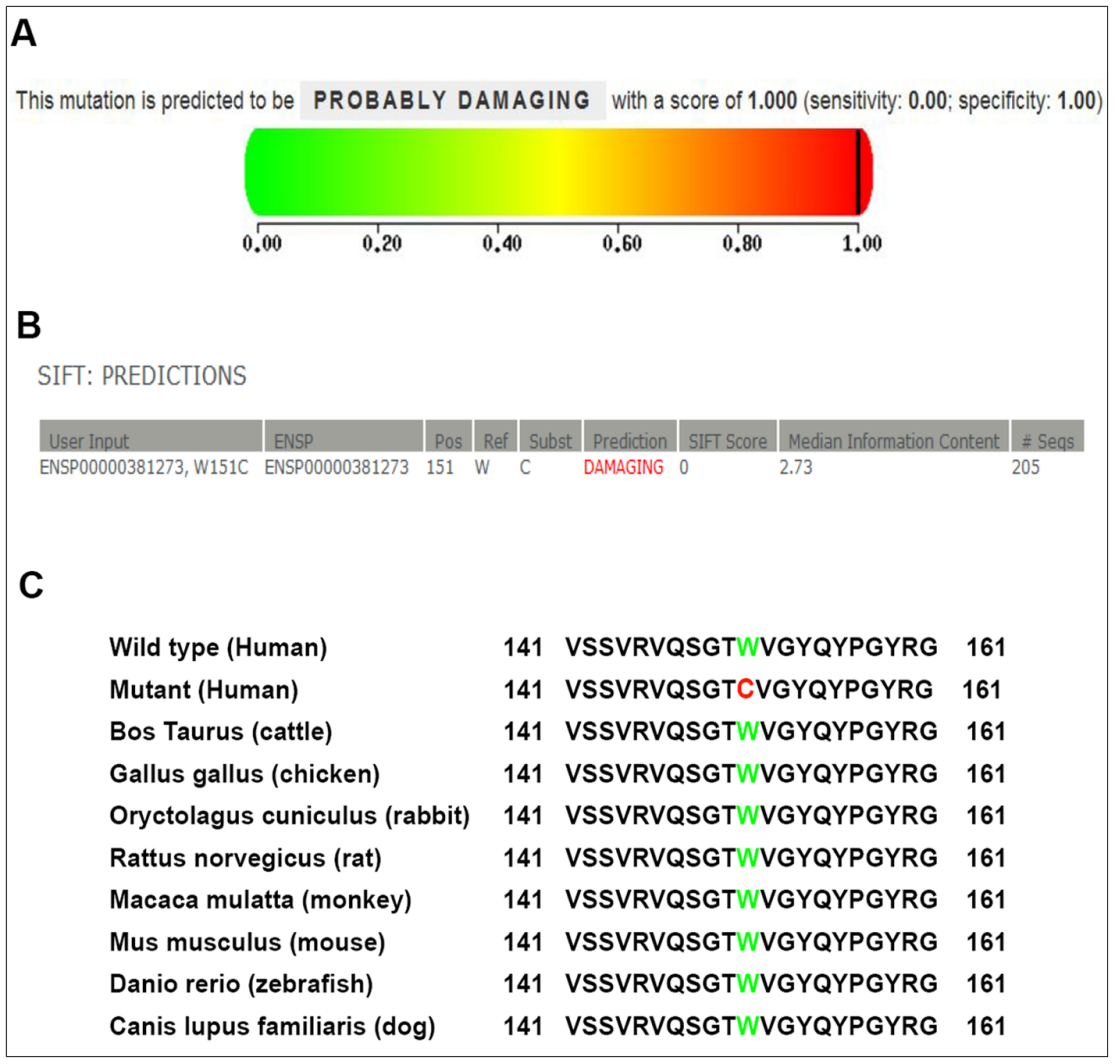
Bioinformatic evaluation of the impact of W151C mutation and multiple-sequence alignment analysis. (A) The PolyPhen-2 program was used to predict the possible impact of the amino acid substitution on the structure and function of βB2-crystallin. The score from PolyPhen-2 analysis is 1.00, which means that the W151C mutation is predicted to probably damage to the structure and function of βB2-crystallin. (B) The outcome from SIFT shows that the amino acid change is probably damaging to the function of protein, with the score is 0.00 and the median information content is 2.73. (C) Multiple-sequence alignment in CRYBB2 from different species reveals that codon 151, where the mutation (p. W151C) occurred, is highly conserved (highlighted in green, the mutant in red).

To determine whether the amino acid substitution induced by W151C mutation would impact the structure and function of βB2-crystallin, the PolyPhen-2 program and SIFT were used. As shown in Figure 5A, the score from PolyPhen-2 analysis was 1.00, which meant that this W151C mutation was predicted to probably damage to the structure and function of βB2-crystallin. Moreover, the prediction result from SIFT also showed that the amino acid change was probably damaging to the function of protein, with the score was 0.00 and the median information content was 2.73 (Figure 5B). All of these results indicated the W151C substitution is likely deleterious and possibly contributes to the disease.

### Multiple-sequence alignment

Using the NCBI websites, a multiple sequence alignment showed that the Tryptophan at position 151 of human CRYBB2 protein (Homo sapiens, NP_000487.1) is highly conserved in various species including Bos taurus (NP_777232.1), Gallus gallus (NP_990506.2), Oryctolagus cuniculus (NP_001082786.1), Rattus norvegicus (NP_037069.1), Macaca mulatta (NP_001116366.1), Mus musculus (NP_031799.1), Danio rerio (NP_001018138.1) and Canis lupus familiaris (NP_001041578.1) (Figure 5C).

### W151C -βB2-crystallin forms nuclear or perinuclear aggregates in HLECs

**Figure 6:**
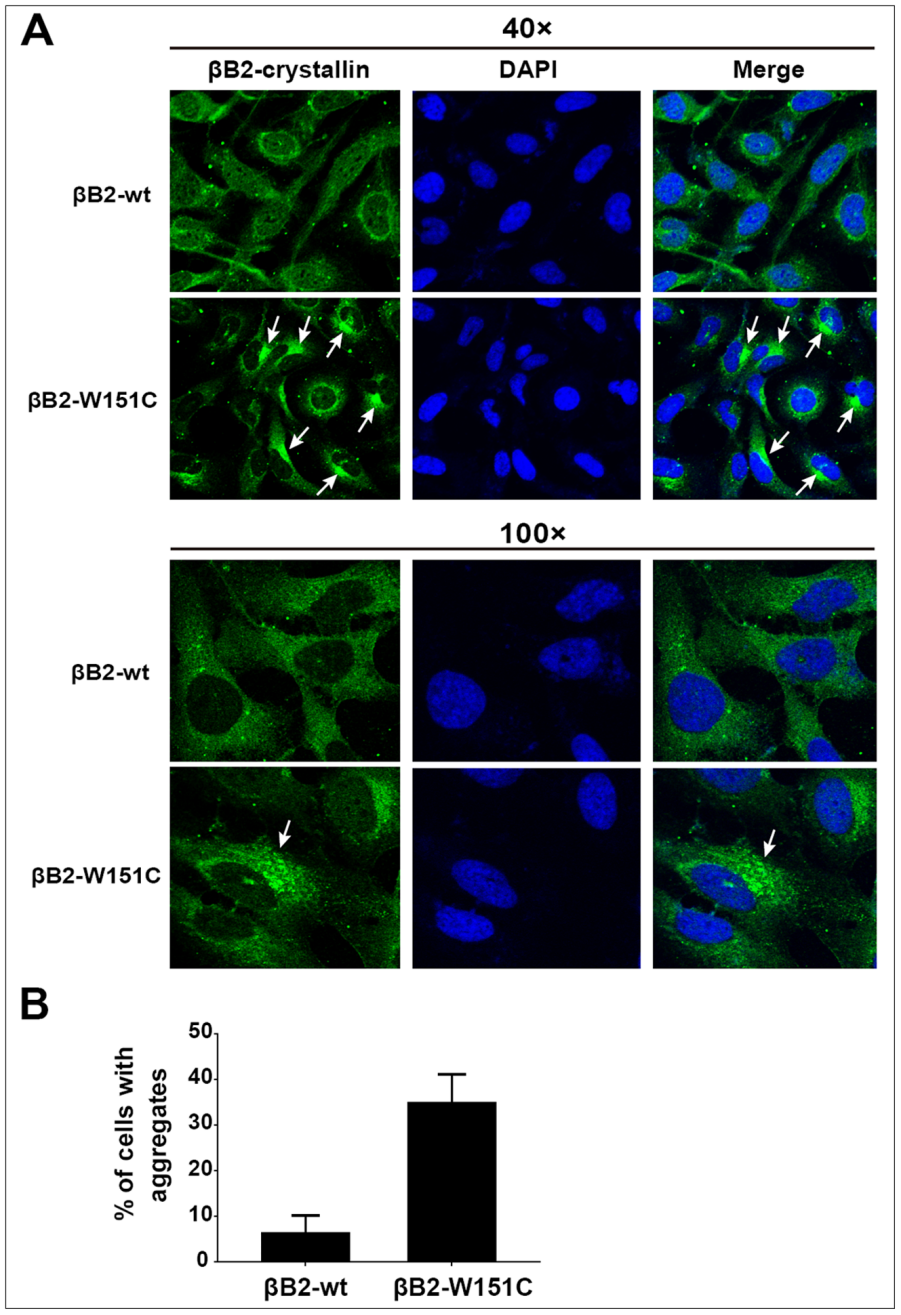
W151C-βB2-crystallin forms aggregates in the cytoplasm and nucleus of HLECs. (A) Representative fluorescence microscopy images of HLECs transfected with GFP-wt-βB2-crystallin or the mutant. Cells transfected with the wt-βB2-crystallin shows a homogenous distribution throughout the cells, while cells transfected with the W151C mutant βB2-crystallin forms aggregates in the perinuclear and nuclear regions. (B) Quantification of the proportion of transfected cells with W151C-βB2-crystallin aggregates. For each experiment, cells containing aggregates were counted in 5 random fields, each containing approximately 30 cells. Each bar represents an average of three independent experiments.

To investigate whether the mutant of βB2-crystallin impacts the solubility and function of βB2-crystallin in cells, GFP-tagged wild-type and the mutant of βB2-crystallin were transfected individually in HLECs. As shown in Figure 6, cells transfected with the wt-βB2-crystallin showed a homogenous distribution throughout the cells and there was a little or no aggregation was observed in cells, indicating that fusing GFP to the crystallin did not perturb the usual solubility of βB2-crystallin. By contrast, approximately 34.7% of cells transfected with the W151C mutant βB2-crystallin formed aggregates of different sizes, mainly in the perinuclear and nuclear regions of the transfected cells. These data indicated that the W151C mutation would damage to the solubility of βB2-crystallin and result in the formation of aggregates in lens cells.

## Discussion

In this study, we identified a missense mutation in exon 6 of CRYBB2 that led to an exchange of Trp for Cys (W151C) in a four-generation Chinese family affected with congenital membranous cataract. This missense variation was found in all the affected family members, but not in unrelated controls or normal family members. Although the identical mutation was reported by Santhiya et al [19] in an Indian family with congenital cataract, the phenotype is very different. The phenotype reported previously in an Indian family was central nuclear cataract, while our Chinese family was membranous cataracts which was a new phenotype related to CRYBB2. We also found that lens opacities in this family appeared after birth and progressed in the early years of life. More interesting, lens upward dislocated and the lens cortex was dissolved gradually with increasing age because of the rupture of capsules. Over 50 years old, cortex of the lens could be dissolved completely. In addition, the mutant W151C of βB2-crystallin would damage to the solubility of βB2-crystallin and result in the formation of aggregates in HLECs.

To date, including W151C, totally fourteen mutations in CRYBB2 had been reported to be associated with congenital cataract [2], [9]-[15]. But the cataract phenotypes in each family were very different despite the identical mutation. In the autosomal dominant congenital cataract, S31W caused coronary cataract [13]. Q155X led to diverse phenotypes, including cerulean [20], coppock-like [21], polymorphic [22], and sutural cataract [23]. D128V resulted in bilateral nuclear cataract surrounded by cortical opacity [24]. V187M mutation showed bilateral anterior axial embryonal nuclear cataract [10]. The morphology of membranous cataract is distinctive from all of these various cataract forms. In addition, the phenotype we reported is distinct from the central nuclear cataract in an Indian family with an identical gene mutation [19]. Membranous cataracts are thin fibrotic lenses caused by the reabsorption of lens proteins. The anterior and posterior lens capsules fuse forming a dense white membrane. Thus, W151C is the first mutation reported for membranous cataract and our Chinese family provided a new phenotype related to CRYBB2.

βB2-crystallin, the major component of β-crystallin, is recognized as a member of the β/γ-crystallin superfamily. Both of β-crystallin and γ-crystallin contain four Greek key motifs. In the β-crystallins, each individual Greek key motif is encoded by separate exon. The β-crystallin gene consists of six exons: the first exon is not translated, the second exon encodes the NH2-terminal extension, and the subsequent four exons are responsible for one Greek key motif each [25]. It is a homodimer at low concentration, and can form a heterodimer with other β-crystallins under physiologic conditions [23]. Each subunit in the homodimer βB2-crystallin includes 16 β-strands, eight in the NH2-terminal domain and eight in the COOH-terminal domain. There are a lot of intermolecular contacts between the NH2-terminal domain and the COOH-terminal domain. Any mutation affecting this intermolecular contact will affect the solubility and stability of CRYBB2, which can destroy the local binding ability, disrupt the dimerization of CRYBB2 protein or impair binding with other lens-soluble proteins. The Q155X mutation, showed partial unfolded structure and decreased structure order, with reduced interactions with other proteins [26]. Another mutation, A188H, located in the β4-sheet, was predicted to impair the dimerization of CRYBB2 protein upon the formation of a new hydrogen bond between histidine and threonine at position149, thereby leading to lens opacity [10]. The D128V mutation was supposed to cause the random coil region between amino acids 126-139 of the mutant protein to become hydrophobic and electropositive [24]. Moreover, W151C mutation which was identified in an Indian family has been predicted to destroy the fourth Greek key motif and increase the protein hydrophobicity. The environment surrounding the amino acid “W” in the wild-type protein is more hydrophilic than the mutant form, which might affect the solubility of the mutant CRYBB2 and hence contribute to cataract formation [19]. In the present study, we demonstrated that expression of the mutant W151C of βB2-crystallin in HLECs led to the formation of intracellular protein aggregates compared to βB2-wt. It is likely that the protein aggregation in the cytoplasm was due to protein conformational changes, which would damage to the solubility of βB2-crystallin and result in the formation of aggregates in cells. Lens clarity depends on regular packing of water soluble proteins. βB2-crystallin as a structural protein plays a key role in maintaining lens transparency. Therefore, this alteration may destroy the microstructure of lens and increase light scattering, leading ultimately to lens opacity.

However, as has been mentioned previously, identical mutation in different families or even the same mutation within the same family can result in radically different cataract morphologies and severities. Conversely, cataracts with similar or identical clinical presentations can result from mutations in completely different genes. The relationship between the genotype and the phenotype of inherited congenital cataracts is still undetermined. This indicated that additional genes or environmental factors might modify the expression of the primary mutation associated with the cataracts. Further studies of this cataract-related genetic defect and the factors that modify their variable phenotypes will improve our understanding of the mechanism of cataract formation and illuminate the developmental biology and biochemistry of the lens.

In summary, the present study described a progressive membranous congenital cataract caused by the W151C mutation of the CRYBB2 gene, expanding the spectrum of phenotypes caused by this mutation. Moreover, this study provided the first evidence for the mutant of βB2-crystallin damages to the solubility of βB2-crystallin and results in the formation of aggregates in cells. However, further studies about cell biology and mutation animal model are necessary to evaluate the precise molecular mechanism caused by the p. Trp151Cys mutation.
